# Extra-Articular Symptoms in Constellation with Selected Serum Cytokines and Disease Activity in Spondyloarthritis

**DOI:** 10.1155/2016/7617954

**Published:** 2016-12-07

**Authors:** Hanna Przepiera-Będzak, Katarzyna Fischer, Marek Brzosko

**Affiliations:** ^1^Department of Rheumatology, Internal Medicine and Geriatrics, Pomeranian Medical University in Szczecin, Unii Lubelskiej 1, 71-252 Szczecin, Poland; ^2^Independent Laboratory of Rheumatic Diagnostics, Pomeranian Medical University in Szczecin, Unii Lubelskiej 1, 71-252 Szczecin, Poland

## Abstract

*Objectives.* In this study, we assessed the extra-articular symptoms in constellation with selected serum cytokines and disease activity in spondyloarthritis (SpA).* Patients and Methods.* We studied 287 SpA patients: 131 had AS, 110 had PsA, and 46 had SAPHO. We assessed extra-articular symptoms in all cases. In 191 SpA patients, we measured serum interleukin-6 (IL-6), interleukin-18 (IL-18), interleukin-23 (IL-23), endothelin-1 (ET-1), vascular endothelial growth factor (VEGF), and epidermal growth factor (EGF).* Results.* Patients with acute anterior uveitis (AAU) had higher VAS (*P* = 0.0008), BADSDAI (*P* = 0.0001), ASDAS-ESR (*P* = 0.04), CRP (*P* = 0.006), IL-6 (*P* = 0.02), and IL-18 (*P* = 0.03) levels. Patients with inflammatory bowel disease (IBD) had higher VAS (*P* = 0.03), CRP (*P* = 0.0009), and IL-6 (*P* = 0.0003) levels. Patients with skin psoriasis had lower VAS (*P* = 0.001) and BASDAI (*P* = 0.00007) levels. Patients with psoriatic onycholysis had lower VAS (*P* = 0.006), BASDAI (*P* = 0.00001), and CRP (*P* = 0.02) and higher IL-23 (*P* = 0.04) levels. Patients with PPP had lower BASDAI (*P* = 0.04) and higher ET-1 (*P* = 0.001) levels.* Conclusions.* SpA patients with increased serum IL-18 and decreased serum ET-1 had an increased risk of extra-articular symptoms. In SpA patients, increased disease activity was associated with an increased risk of AAU and IBD and a decreased risk of skin psoriasis, psoriatic onycholysis, and PPP.

## 1. Introduction

Ankylosing spondylitis (AS), psoriatic arthritis (PsA), and SAPHO syndrome (SAPHO) are seronegative spondyloarthropathies (SpA) which are connected with the presence of HLA-B27 antigen [1–3]. In the course of the diseases different extra-articular clinical symptoms could appear, such as acute anterior uveitis AAU, inflammatory bowel disease (IBD), psoriasis, psoriatic onychopathy, and palmoplantar pustulosis (PPP) [1–3]. The presence of different extra-articular symptoms in the course of SpA has an influence on the course of SpA and therapeutic decisions [4–9].

Several proinflammatory cytokines such as interleukin-6 (IL-6), interleukin-18 (IL-18), interleukin-23 (IL-23) are considered to play a role in pathogenesis of SpA [10–14]. Also cytokines involved in angiogenesis such as vascular endothelial growth factor (VEGF), epidermal growth factor (EGF), and proinflammatory peptide endothelin-1 (ET-1) are considered to be involve in inflammatory process in SpA [15–17].

There are data that development of different extra-articular symptoms is connected with elevated levels of different markers of inflammatory process [4, 6, 13]. Additionally vascular abnormalities and endothelial dysfunction are considered to play a role in the pathogenesis of AAU, IBD, and psoriasis [18–20].

## 2. Objectives

The aim of our study was to assess extra-articular symptoms in constellation with selected serum cytokines and disease activity in SpA. We selected cytokines that play a role in the pathogenesis of SpA and are involved in angiogenesis and endothelial function.

## 3. Materials and Methods

This study was approved by the Ethics Committee of the Pomeranian Medical University in Szczecin (KB-0012/106/10; 27SEP2010). Informed consent was obtained from all patients. We studied 287 SpA patients: 131 had AS, 110 had PsA, and 46 had SAPHO. All patients were Caucasian. The following data were recorded: age, sex, disease duration, and extra-articular symptoms: acute anterior uveitis (AAU), inflammatory bowel disease (IBD), skin psoriasis, psoriatic onychopathy, and positivity for HLA B27. The diagnosis of AS was made according to modified New York criteria [1]. The diagnosis of PsA was made according to the Caspar classification criteria [2]. The diagnosis of SAPHO syndrome was made according to the Kahn criteria [3]. In the PsA group, skin changes were assessed according to the Psoriasis Area and Severity Index (PASI) [21]. The patient's pain due to the disease at the time of examination was assessed using a visual analogue scale (VAS).

We also assessed the Bath Ankylosing Spondylitis Disease Activity Index (BASDAI). This index has a possible score of 0–10, with a higher score indicating greater disease activity. We regarded patients as active if the BASDAI score was > 4 [22].

The Ankylosing Spondylitis Disease Activity Score (ASDAS) was assessed using ESR. The ASDAS-ESR was calculated, in AS patients and PsA patients with axial joint involvement, using online calculator available at the Assessment of Spondyloarthritis International Society website.

Disease activity score calculation, in PsA patients, were made use of free online Disease Activity Score 28 (DAS28) calculator.

In the first 191 SpA patients, 81 had AS, 76 had PsA, and 34 had SAPHO; we studied serum levels of selected cytokines: IL-6, IL-23, IL-18, endothelin-1(ET-1), VEGF, and EGF. Additionally, we studied CRP and ESR. The controls were 30 healthy volunteers. The methods of assessment of these cytokines were presented in our previous paper [13].

Data distributions were assessed using the Kolmogorov–Smirnov test. Data are described as mean ± standard deviation and median (Q1 and Q3). The *R* values of correlations were determined and corresponding *P* values <0.05 were considered significant. The groups were compared using Student's *t*-test, Mann–Whitney *U* test, and Kruskal-Wallis test. To assess parameters Pearson's chi-squared test (*χ* ^2^), logistic regression analysis, and step-wise analysis were performed. The level of significance was set at *P* < 0.05. The statistical analysis was performed using STATISTICA version 8.0, StatSoft, Inc., Tulsa, United States.

## 4. Results

The clinical characteristics of SpA patients and the prevalence of extra-articular symptoms are presented in Table 1. Data presenting serum levels of selected cytokines and markers of disease activity in subgroup of 191 SpA patients and 30 controls are presented in our previous paper by Przepiera-Będzak et al. [13].

### 4.1. Acute Anterior Uveitis

AAU was observed in 12.1% of SpA patients, in 26.0% of AS, and in 0.9% of PsA. No one patient with SAPHO had AAU (Table 1). The prevalence of AAU was higher in AS than in PsA (*P* < 0.000001) and in AS than in SAPHO (*P* = 0.0001). We compared SpA patients with AAU to those without AAU. SpA patients with AAU had longer disease duration (*P* = 0.0002) and higher prevalence of HLA-B27 antigen (*P* = 0.0001). SpA patients with AAU had higher disease activity as assessed using the VAS (*P* = 0.0008), BADSDAI (*P* = 0.0001), ASDAS-ESR (*P* = 0.04), and CRP (*P* = 0.006). Additionally, they had lower disease activity as assessed using the DAS28 (*P* < 0.0001) (Table 2).

In SpA patients increased CRP (*P* = 0.01) and BASDAI (*P* = 0.005) were associated with increased risk of AAU (Table 4). SpA patients with AAU compared to those without AAU had higher serum IL-6 (*P* = 0.02) and IL-18 (*P* = 0.03) levels (Table 2). SpA patients with AAU compared to healthy controls had higher serum IL-6 levels (*P* = 0.02). SpA patients with AAU compared to healthy controls had higher serum IL-18 levels (*P* = 0.00009). In SpA patients as compared to healthy controls, increased serum levels of IL-6 (*P* = 0.02), IL-23 (*P* = 0.03), and Il-18 (*P* = 0.0006) were associated with increased risk of AAU (Table 5). In SpA patients as compared to healthy controls, decreased serum levels of ET-1 (*P* = 0.0007) were associated with increased risk of AAU (Table 5).

### 4.2. Inflammatory Bowel Disease

IBD was present in 3.5% SpA patients and in 7.6% AS patients. No one patient with PsA or SAPHO had IBD (Table 1). The prevalence of IBD was higher in AS than in PsA patients (*P* = 0.004). We compared SpA patients with IBD to those without IBD. SpA patients with IBD had higher prevalence of HLA-B27 antigen (*P* = 0.04) and higher disease activity as assessed using the VAS (*P* = 0.03), CRP (*P* = 0.0009), and IL-6 (*P* = 0.0003). Additionally, they had lower disease activity as assessed using the DAS28 (*P* < 0.0001) (Table 2). SpA patients with IBD compared to healthy controls had higher serum IL-6 levels (*P* = 0.007). When SpA patients were compared to controls, increased serum levels IL-18 (*P* = 0.03) were associated with an increased risk of IBD (Table 5).

### 4.3. Skin Psoriasis

Skin psoriasis was present in 32.4% of SpA patients, in 82.7% PsA patients, and in 1.5% AS patients (Table 1). We compared SpA patients with skin psoriasis to those without skin psoriasis. SpA patients with skin psoriasis had shorter disease duration (*P* = 0.004), lower prevalence of HLA-B27 antigen (*P* = 0.00004), and lower disease activity as assessed using the VAS (*P* = 0.001), and BASDAI (*P* = 0.00007) (Table 3). In SpA patients, increased VAS (*P* = 0.003) and BASDAI (*P* = 0.002) were associated with decreased risk of skin psoriasis (Table 4).

There were no differences in serum levels of IL-6, IL-23, IL-18, VEGF, and EGF between SpA patients with and without skin psoriasis (Table 3). There were no correlations between serum IL-6, IL-23, IL-18, and PASI score (all *P* > 0.05). In SpA patients as compared to healthy controls, increased serum levels of IL-18 (*P* = 0.0002) and decreased serum levels of ET-1 (*P* = 0.006) were associated with increased risk of skin psoriasis (Table 5).

### 4.4. Psoriatic Onychopathy

Psoriatic onychopathy was present in 25.4% SpA patients and 66.4% PsA patients (Table 1).

We compared SpA patients with psoriatic onychopathy to those without psoriatic onychopathy. SpA patients with psoriatic onychopathy had shorter disease duration (*P* = 0.009), lower prevalence of HLA-B27 antigen (*P* = 0.0004), and lower disease activity as assessed using the VAS (*P* = 0.006), BASDAI (*P* = 0.00001), CRP (*P* = 0.02), and IL-23 (*P* = 0.04) (Table 3).

SpA patients with psoriatic onychopathy compared to healthy controls had higher IL-23 level (*P* = 0.02).

SpA patients with increased BASDAI (*P* = 0.002) had lower risk of psoriatic onychopathy (Table 4).

In SpA patients, compared to healthy controls, increased serum levels of IL-18 (*P* = 0.0002) and decreased serum levels of ET-1 (*P* = 0.008) were associated with increased risk of psoriatic onychopathy (Table 5).

### 4.5. Palmoplantar Pustulosis

PPP was present in 14.6% SpA patients and in 91.3% SAPHO patients (Table 1). We compared SpA patients with PPP with those without PPP. SpA patients with PPP had shorter disease duration (*P* = 0.0009), lower prevalence of HLA-B27 antigen (*P* = 0.0008), and lower disease activity as assessed using the BASDAI (*P* = 0.04). They had higher ESR (*P* = 0.004) and higher serum ET-1 (*P* = 0.001) (Table 3). There were no differences in serum ET-1 between SpA patients with PPP and healthy controls (*P* = 0.4). In SpA patients compared to healthy controls increased serum levels of IL-18 (*P* = 0.01) were associated with increased risk of PPP (Table 5).

## 5. Discussion

We presented extra-articular symptoms in constellation with disease activity and selected serum cytokines which are involved in disease activity, angiogenesis, and endothelial function in SpA patients.

The percentage of AAU in SpA patients and AS patients in our study was similar to those presented by other authors [4–6, 9].

The most important genetic factor associated with AAU is HLA-B27 [8]. We confirmed these associations in our study too.

In our study AAU was the most frequent in AS patients. Additionally, SpA patients with AAU had higher levels of ASDAS-ESR and lower DAS28. These confirm observations of other authors that AAU is more frequent in axial SpA [6, 8, 9]. These suggest that there is association between severity of axial, but not peripheral arthritis with AAU occurrence in SpA.

We also confirmed as other authors that patients with AAU had longer disease duration and higher disease activity [4, 8].

IL-6 plays role in arthritis but its role in SpA pathogenesis is controversial. Kramer et al. [23] presented increased serum levels of IL-6 in patients with active uveitis. In our study we found that SpA patients with AAU had higher serum levels of IL-6. Additionally, increased serum IL-6 was associated with increased risk of AAU in SpA. In our previous study by Przepiera-Będzak et al. [14] we showed increased serum IL-6 in SpA patients and positive correlation with markers of disease activity such as ESR and CRP. These could suggest the role of IL-6 in pathogenesis of AAU in SpA.

IL-18 is considered as one of proinflammatory cytokine which also activate and deregulate endothelial function. In our previous study we confirmed higher levels of IL-18 in SpA patients and AS patients [13]. In current study we additionally confirmed that serum IL-18 was higher in SpA patients with AAU and compared to healthy controls. Additionally, increased serum IL-18 increased risk of AAU. These suggest the role of IL-18 in the pathogenesis of AAU in SpA by influence on inflammatory process and endothelial function.

The percentage of IBD in SpA patients in our study was similar to those presented by other authors [4, 5]. Patients with IBD had longer disease duration and higher disease activity, which correspond with other authors observations [6]. Additionally we observed that SpA patients with IBD had lower DAS 28. This confirmed data presented by others that IBD is less frequent in SpA patients with peripheral arthritis [6]. These suggest that there is association between severity of axial, but not peripheral arthritis and IBD occurrence in SpA.

The percentage of SpA patients with skin psoriasis in our study was similar to the results presented by others [6, 9]. Peluso et al. [7] presented that 89.2% of PsA patients had psoriasis, and that PsA patients with skin psoriasis had longer disease duration and more frequent axial disease. In our study the percentage of PsA patients with psoriasis was the same, but data concerning disease activity and disease duration were reversible. We found that patients with skin psoriasis had shorted disease duration and lower ASDAS-ESR which suggest that they had lower activity of axial SpA. On the other hand Essers et al. [4] in study of 216 AS patients found that history of psoriasis was associated with grater age and lower CRP. In our study we found the same associations in SpA patients with skin psoriasis. Additionally, we confirmed that increased disease activity measured by VAS and BASDAI decreased risk of skin psoriasis. These suggest that there is no association between severity of axial and peripheral arthritis and skin psoriasis occurrence in SpA.

We also found that SpA patients with skin psoriasis, compared to SpA group without that symptom, had lower levels of proinflammatory cytokines such as IL-6, IL-23, IL-18, VEGF, and EGF. Nevertheless, we confirmed that increased serum IL-18 levels were associated with increased risk of skin psoriasis in SpA. These foundlings suggest that some markers of disease activity did not influence on prevalence of psoriatic skin changes. On the other hand it can confirm the role of IL-18 in pathogenesis of skin psoriasis by influence on disease activity and endothelial function.

IL-23 is produced by keratinocytes and is considered to play a role in SpA pathogenesis. In our previous study by Przepiera-Będzak et al. [14] we showed increased serum IL-23 levels in SpA patients. It was interesting that we did not find correlation between serum IL-23 and PASI [14]. In current study there were no differences in serum level of IL-23 between patients with and without psoriasis, which suggested that serum IL-23 could influence pathogenesis of skin psoriasis but did not reflect a presence of skin disease in SpA patients.

Psoriatic onychopathy was observed only in PsA group. Peluso et al. have presented psoriatic onychopathy in 89.2% PsA patients [7]. In SpA patients 66.4% had psoriatic onychopathy. In our study we fund that longer disease duration and higher disease activity were associated with decreased risk of psoriatic onychopathy. These suggest that there is no association between severity of axial and peripheral joints arthritis and psoriatic onychopathy occurrence in SpA.

The axis IL-17/ILK-23 is considered to play the crucial role in SpA pathogenesis. In our study we presented association between psoriatic onychopathy and serum IL-23. SpA patients with psoriatic onychopathy had higher serum IL-23 than healthy controls. In our previous study by Przepiera-Będzak et al. [14] we confirmed that SpA patients had higher serum IL-23 than healthy controls. Results of our study confirm the role IL-23 in pathogenesis of psoriatic onychopathy.

There were data which confirmed increased serum IL-18 in SAPHO. These suggested role of IL-18 in pathogenesis of SAHO [12, 13]. In our study increased serum IL-18 was associated with increased risk of PPP. This could confirm the role of IL-18 in pathogenesis of PPP by influence on disease activity and endothelial function.

ET-1 plays a role in inflammation and vasculopathy. Kuryliszyn-Moskal et al. [24] presented increased serum ET-1 in rheumatoid arthritis patients with extra-articular symptoms. In a previous study by Przepiera-Będzak et al. [13], serum ET-1 levels were lower in SpA patients than in controls, but levels were significantly higher in SAPHO than in AS or PsA. In our current study SpA patients with PPP compared with those without PPP had higher ET-1 level. So we can speculate that maybe ET-1 by its influence on vasculopathy and inflammation can play a role in PPP pathogenesis in SpA patients.

## 6. Conclusion

The analysis of serum levels of selected cytokines in SpA patients with and without extra-articular symptoms and healthy controls confirmed the following:

- patients with AAU had higher serum IL-6 and IL-18;
- patients with IBD had higher levels of IL-6;
- patients with nail psoriasis had higher levels of IL-23;
- patients with PPP had higher levels of ET-1.

In SpA patients, increased serum IL-18 and decreased serum ET-1 were associated with an increased risk of extra-articular symptoms. Additionally, increased serum IL-6 and IL-23 increased the risk of AAU. Among SpA patients, increased disease activity was associated with an increased risk of AAU and IBD and a decreased risk of skin psoriasis, psoriatic onycholysis, and PPP.

## Figures and Tables

**Table 1:** The clinical characteristics and the percentage of extra-articular symptoms of spondyloarthritis patients.

	Total (*n* = 287) *N* ± SD *N* (%)	AS (*n* = 131) *N* ± SD *N* (%)	PsA (*n* = 110) *N* ± SD *N* (%)	SAPHO (*n* = 46) *N* ± SD *N* (%)
%	100	45,6	38,3	16,0
Age (age)	49.2 ± 12.8	47.3 ± 13.2	50.7 ± 12.5	51.0 ± 12.1
Sex	130 F, 157 M	28 F, 103 M	62 F, 48 M	40 F, 6 M
Disease duration (years)	8.7 ± 8.2	13.3 ± 9.2	5.8 ± 4.9	2.9 ± 2.6
AAU, *n* (%)	35 (12.1)	34 (26.0)	1 (0.9)	0 (0)
IBD (any), *n* (%)	10 (3.5)	10 (7.6)	0 (0)	0 (0)
Amyloidosis, *n* (%)	3 (1,1)	2 (1,5)	1 (0.9)	0 (0)
Psoriasis, *n* (%)	93 (32.4)	2 (1.5)	91 (82.7)	0 (0)
Psoriatic onycholysis, *n* (%)	73 (25.4)	0 (0)	73 (66.4)	0 (0)
Only skin psoriasis without nail involvement, *n* (%)	62 (21.6)	2 (1.5)	60 (54.5)	0 (0)
Only psoriatic onycholysis without skin involvement, *n* (%)	31 (10.8)	0 (0)	31 (28.2)	0 (0)
Palmoplantar pustulosis, *n* (%)	42 (14.6)	0 (0)	0 (0)	42 (91.3)
Acne, *n* (%)	3 (1.1)	0 (0)	0 (0)	3 (6.5)
Primary biliary cirrhosis, *n* (%)	1 (0.3)	1 (0.8)	0 (0)	0 (0)
HLA B-27 (positive/done)	105/165	88/91	13/51	4/23

AAU: acute anterior uveitis, AS: ankylosing spondylitis, IBD: inflammatory bowel disease, *N*: number of patients, PsA: psoriatic arthritis, SAPHO: synovitis acne pustulosis hyperostosis osteitis syndrome, and SD: standard deviation.

**Table 2:** Comparison of serum levels of selected cytokines and markers of disease activity in spondyloarthritis patients with and without acute anterior uveitis and inflammatory bowel disease.

	AAU present	AAU absent	*P*	IBD present	IBD absent	*P*
Age (years)	44.8 ± 12.1	48.9	0.06	42.6 ± 11.4	48.3 ± 13.1	0.1
Disease duration (years)	10.0 (5.0, 19.0)	5.0 (2.0, 9.0)	0.0002	10.0 (5.0, 13.5)	5.0 (2.0, 10.)	0.06
HLA-B27	94.4 ± 23.5	47.1	0.0001	100	62	0.04
VAS pain (mm)	63.7 ± 28.3	49.4 ± 23.8	0.0008	67.5 ± 28.7	51.4 ± 24.8	0.03
BASDAI	5.8 ± 2.8	3.9 ± 2.7	0.0001	5.3	4.2	0.1
ASDAS-ESR	2.7 (2.2, 3.4)	2.3 (1.8, 3.0)	0.04	3.1 (2.2, 4.1)	2.4 (1.8, 3.2)	0.06
DAS28	0.0	4.24	<0.0001	0.0	4.24	<0.0001
CRP (mg/L)	10.5 (2.6, 21.1 0)	5.0 (2.0, 11.4)	0.006	19.0 (7.8, 31.1)	5.5 (2.1, 12.3)	0.0009
ESR (mm/h)	14.0 (5.0, 35.)	13.5 (6.0, 24.5)	0.4	25.5	14.0	0.05
IL-6 (pg/mL)	4.5 (1.5, 10.1)	2.9 (1.5, 6.5)	0.02	3.3 (1.5, 6.4)	2.9 (1.5, 20.7)	0.0003
IL-18 (pg/mL)	323.5 (228.7, 454.5)	271.8 (203.5, 346.2)	0.03	333.8 (237.5, 285.2)	271.8 (207.3, 373.9)	0.4
IL-23(pg/mL)	0.0 (0.0, 2.8)	0.0 (0.0, 1.8)	0.2	0.0 (0.0, 0.3)	0.0 (0.0, 2.0)	0.2
VEGF (pg/mL)	406.2 (270.0, 880.0)	343.1 (223.2, 545.7)	0.05	341.3 (206.3, 505.0)	347.4 (225.9, 562.2)	0.3
EGF (pg/mL)	115.0 (62.0, 182.0)	104.0 (68.0, 168.0)	0.5	81.0 (32.0, 141.0)	104.0 (68.0, 182.0)	0.2
ET-1 (pg/mL)	1.1 ± 0.4	1.3 ± 0.6	0.07	1.3 ± 0.5	1.3 ± 0.5	0.3

Data are presented as mean ± standard deviation, median (Q1 and Q3). AAU: acute anterior uveitis, ASDAS-ESR: Ankylosing Spondylitis Disease Activity Score, BASDAI: Bath Ankylosing Spondylitis Disease Activity Index, CRP: C-reactive protein, DAS28: Disease Activity Score 28, EGF: epidermal growth factor, ESR: erythrocyte sedimentation rate, ET-1: endothelin-1, IBD: inflammatory bowel disease, IL-6: interleukin-6, IL-18: interleukin-18, IL-23: interleukin-23, VAS pain: visual analogue scale of patient's pain, and VEGF: vascular endothelial growth factor.

**Table 3:** Comparison of serum levels of selected cytokines and markers of disease activity in spondyloarthritis patients with and without skin psoriasis, psoriatic onycholysis, and palmoplantar pustulosis.

	Psoriasis present	Psoriasis absent	*P*	Psoriatic onycholysis present	Psoriatic onycholysis absent	*P*	PPP present	PPP absent	*P*
Age (years)	52.5 ± 12.5	46.5 ± 13.0	0.001	50.1 ± 14.2	47.7 ± 12.7	0.1	52.4 ± 11.9	47.5 ± 13.2	0.03
Disease duration (years)	4.0 (2.0, 8.0)	6.0 (2.0, 11.0)	0.004	4.0 (2.0, 7.0)	6.5 (2.0, 12.0)	0.009	2.0 (1.0, 5.0)	6.0 (3.0, 11.0)	0.00009
HLA-B27	22	71	0.00004	28	70	0.0004	23	68	0.0008
VAS pain (mm)	43.1 ± 21.0	55.1 ± 25.6	0.001	44.0 ± 22.2	54.2 ± 25.4	0.006	48.6 ± 20.8	51.9 ± 25.6	0.3
BASDAI	3.1 ± 2.4	4.7 ± 2.8	0.00007	2.8 ± 2.5	4.7 ± 2.7	0.00001	4.3 ± 2.6	4.2 ± 2.8	0.04
ASDAS-ESR	1.8 (1.6, 2.1)	2.5 (1.9, 3.2)	0.006	1.9 (1.6, 2.4)	2.5 (1.8, 3.2)	0.06	—	—	—
DAS28	4.25 (3.8, 4.5)	4.05 (3.0, 6.9)	0.3	4.24 (3.8, 4.5)	4.28 (3.7, 4.8)	0.3	—	—	—
CRP (mg/L)	4.1 (1.6, 9.4)	6.35 (2.4, 13.3)	0.07	3.8 (1.6, 9.6)	6.1 (2.1, 13.5)	0.02	4.5 (1.1, 12.4)	5.6 (2.4, 12.0)	0.4
ESR (mm/h)	13.0 (6.0, 22.0)	14.0 (6.0, 28.0)	0.2	13.0 (7.0, 22.0)	14.0 (6.0, 27.0)	0.2	21.0 (10.5, 36.0)	13.0 (6.0, 24.0)	0.004
IL-6 (pg/mL)	2.5 (1.4, 6.2)	3.6 (1.7, 7.9)	0.4	3.4 (1.4, 6.5)	3.3 (1.6, 6.7)	0.2	2.4 (1.0, 6.6)	3.4 (1.5, 6.7)	0.2
IL-18 (pg/mL)	290.1 (225.4, 373.9)	259.7 (205.3, 372.3)	0.3	286.0 (225.4, 408.9)	264.9 (202.9, 370.0)	0.4	265.0 (193.3, 377.9)	278.2 (213.6, 373.1)	0.4
IL-23(pg/mL)	0.0 (0.0, 0.9)	0.0 (0.0, 2.5)	0.3	0.0 (0.0, 0.9)	0.0 (0.0, 2.5)	0.04	0.0 (0.0, 0.3)	0.0 (0.0, 2.5)	0.2
VEGF (pg/mL)	343.5 (211.7, 680.0)	348.9 (229.6, 500.0)	0.5	289.6 (205.7, 759.7)	347.8 (260.0, 500.0)	0.5	333.1 (235.3, 390.0)	348.9.3 (225.9, 612.1)	0.1
EGF (pg/mL)	130.0 (74.0, 196)	95.0 (68.0, 160.0)	0.1	120.0 (74.0, 196.0)	103.0 (66.0, 164.0)	0.23	104.0 (58.0, 168)	107.0 (68.0, 182.0)	0.4
ET-1 (pg/mL)	1.3 ± 0.6	1.2 ± 0.5	0.5	1.21 ± 0.4	1.15 ± 0.6	0.5	1.5 ± 0.7	1.2 ± 0.5	0.001

Data are presented as mean ± standard deviation, median (Q1 and Q3). AAU: acute anterior uveitis, ASDAS-ESR: Ankylosing Spondylitis Disease Activity Score, BASDAI: Bath Ankylosing Spondylitis Disease Activity Index, CRP: C-reactive protein, DAS28: Disease Activity Score 28, EGF: epidermal growth factor, ESR: erythrocyte sedimentation rate, ET-1: endothelin-1, IBD: inflammatory bowel disease, IL-6: interleukin-6, IL-18: interleukin-18, IL-23: interleukin-23, PPP: palmoplantar pustulosis, VAS pain: visual analogue scale of patient's pain, and VEGF: vascular endothelial growth factor.

**Table 4:** A logistic regression model of the OR of selected markers of disease activity in spondyloarthritis groups of patients with different extra-articular symptoms compared to controls.

Covariates	AAU		IBD		Psoriasis		Psoriatic onycholysis		PPP	
	OR (95% CI)	*P*	OR (95% CI)	*P*	OR (95% CI)	*P*	OR (95% CI)	*P*	OR (95% CI)	*P*
Disease duration ≥ 5 years	4.05 (1.62–10.12)	0.002	3.7 (0.72–18.84)	0.11	0.58 (0.26–1.25)	0.16	0.29 (0.15–0.59)	0.0004	0.22 (0.09–0.53)	0.0007
VAS ≥ 40 mm	2.28 (0.91–5.7)	0.07	2.22 (0.43–11.30)	0.33	0.31 (0.14–0.68)	0.003	0.67 (0.33–1.36)	0.2	1.22 (0.49–3.08)	0.65
BASDAI ≥ 4	3.56 (1.46–8.66)	0.005	3.10 (0.57–16.86)	0.18	0.24 (0.10–0.61)	0.002	0.33 (0.16–0.67)	0.002	0.61 (0.25–1.47)	0.27
CRP ≥ 10 mg/L	2.90 (1.26–6.67)	0.01	4.03 (0.93–17.51)	0.06	0.52 (0.21–1.29)	0.16	0.59 (0.28–1.24)	0.16	1.05 (0.44–2.49)	0.9

AAU: acute anterior uveitis, BASDAI: Bath Ankylosing Spondylitis Disease Activity Index, CRP: C-reactive protein, IBD: inflammatory bowel disease, OR: odds ratio, PPP: palmoplantar pustulosis, and VAS pain: visual analogue scale of patient's pain.

**Table 5:** A logistic regression model of the OR of serum levels of selected cytokines in spondyloarthritis groups of patients with different extra-articular symptoms compared to controls.

Covariates	AAU		IBD		Psoriasis		Psoriatic onycholysis		PPP	
	OR (95% CI)	*P*	OR (95% CI)	*P*	OR (95% CI)	*P*	OR (95% CI)	*P*	OR (95% CI)	*P*
IL-6 ≥ 6.64 pg/mL	6.75 (1.23–36.91)	0.02	5.4 (0.61–47.76)	0.12	4.29 (0.81–22.80)	0.08	4.35 (0.87–21.64)	0.07	5.06 (0.91–28.15)	0.06
IL-18 ≥ 227.45 pg/mL	8.17 (2.46–27.03)	0.0006	7.0 (1.17–41.53)	0.03	9.0 (2.87–28.16)	0.0002	6.82 (2.50–18.59)	0.0002	3.69 (1.27–10.71)	0.01
IL-23 ≥ 2.5 pg/mL	23.9 (1.28–444.01)	0.03	13.6 (0.49–373.39)	0.12	15.6 (0.83–291.96)	0.06	11.49 (0.63–209.44)	0.09	7.55 (0.34–166.21)	0.19
ET-1 ≤ 1,081 pg/mL	9.45 (2.56–34.79)	0.0007	0.23 (0.04–1.11)	0.06	5.77 (1.65–20.15)	0.006	4.93 (1.50–16.20)	0.008	1.89 (0.49–7.29)	0.35
VEGF ≥ 420.9 pg/mL	2.5 (0.60–10.26)	0.20	0.83 (0.07–9.68)	0.8	1.18 (0.34–4.07)	0.7	1.71 (0.51–5.82)	0.38	0.5 (0.08–2.99)	0.4
EGF ≥ 172.0 pg/mL	1.28 (0.27–5.93)	0.7	0.33 (0.01–7.23)	0.4	1.34 (0.36–4.94)	0.6	1.34 (0.36–4.94)	0.6	0.96 (0.18–4.92)	0.9

AAU: acute anterior uveitis, EGF: epidermal growth factor, ET-1: endothelin-1, IBD: inflammatory bowel disease, IL-6: interleukin-6, IL-18: interleukin-18, IL-23: interleukin-23, OR: odds ratio, PPP: palmoplantar pustulosis, and VEGF: vascular endothelial growth factor.
